# Identification and validation of putative biomarkers by in silico analysis, mRNA expression and oxidative stress indicators for negative energy balance in buffaloes during transition period

**DOI:** 10.5713/ab.23.0284

**Published:** 2024-01-20

**Authors:** Savleen Kour, Neelesh Sharma, Praveen Kumar Guttula, Mukesh Kumar Gupta, Marcos Veiga dos Santos, Goran Bacic, Nino Macesic, Anand Kumar Pathak, Young-Ok Son

**Affiliations:** 1 Division of Veterinary Medicine, Faculty of Veterinary Sciences & Animal Husbandry, Sher-e-Kashmir University of Agricultural Sciences & Technology of Jammu, R.S. Pura, Jammu, UT of J&K 181 102, India; 2 Department of Biotechnology and Medical Engineering, National Institute of Technology Rourkela, Odisha 769 008, India; 3 Department of Animal Sciences, School of Veterinary Medicine and Animal Sciences, University of São Paulo, Pirassununga, SP 13635-900, Brazil; 4 Clinic for Reproduction and Theriogenology, Faculty of Veterinary Medicine, University of Zagreb, Zagreb 100 00, Croatia; 5 Division of Animal Nutrition, Faculty of Veterinary Sciences & Animal Husbandry, Sher-e-Kashmir University of Agricultural Sciences & Technology of Jammu, R.S. Pura, Jammu, UT of J&K 181 102, India; 6 Department of Animal Biotechnology, Faculty of Biotechnology, College of Applied Life Sciences and Interdisciplinary Graduate Program in Advanced Convergence Technology and Science, Jeju National University, Jeju 690756, Korea

**Keywords:** Beta-hydroxy Butyric Acid (BHBA), Buffaloes, Gene Expression, In-silico, Ketosis, Oxidative Stress Markers

## Abstract

**Objective:**

Transition period is considered from 3 weeks prepartum to 3 weeks postpartum, characterized with dramatic events (endocrine, metabolic, and physiological) leading to occurrence of production diseases (negative energy balance/ketosis, milk fever etc). The objectives of our study were to analyze the periodic concentration of serum beta-hydroxy butyric acid (BHBA), glucose and oxidative markers along with identification, and validation of the putative markers of negative energy balance in buffaloes using in-silico and quantitative real time-polymerase chain reaction (qRT-PCR) assay.

**Methods:**

Out of 20 potential markers of ketosis identified by in-silico analysis, two were selected and analyzed by qRT-PCR technique (upregulated; acetyl serotonin o-methyl transferase like and down regulated; guanylate cyclase activator 1B). Additional two sets of genes (carnitine palmotyl transferase A; upregulated and Insulin growth factor; downregulated) that have a role of hepatic fatty acid oxidation to maintain energy demands via gluconeogenesis were also validated. Extracted cDNA (complementary deoxyribonucleic acid) from the blood of the buffaloes were used for validation of selected genes via qRT-PCR. Concentrations of BHBA, glucose and oxidative stress markers were identified with their respective optimized protocols.

**Results:**

The analysis of qRT-PCR gave similar trends as shown by in-silico analysis throughout the transition period. Significant changes (p<0.05) in the levels of BHBA, glucose and oxidative stress markers throughout this period were observed. This study provides validation from in-silico and qRT-PCR assays for potential markers to be used for earliest diagnosis of negative energy balance in buffaloes.

**Conclusion:**

Apart from conventional diagnostic methods, this study improves the understanding of putative biomarkers at the molecular level which helps to unfold their role in normal immune function, fat synthesis/metabolism and oxidative stress pathways. Therefore, provides an opportunity to discover more accurate and sensitive diagnostic aids.

## INTRODUCTION

Immediately before calving and during first few weeks of lactation, there is increased mammary gland activity and lipid mobilization in the body which eventually causes energy deprivation in dairy cattle [1]. Despite having homeostatic mechanism to maintain the basal levels of metabolic parameters, changes in their concentration and activity occur because of pregnancy and lactation demand. These changes are not necessarily indicative of disease condition but makes cattle physiologically unstable during transition period. This instability makes cattle susceptible to several metabolic disorders which eventually compromises their productivity [2]. Dairy animals face more oxidative stress during early lactation or just after parturition than advanced pregnant cattle, and this appears to be the reason for their increased susceptibility to production diseases (e.g. mastitis, metritis, retention of fetal membranes etc.) [3]. With the progress of pregnancy, lipid peroxidation (LPO) level (oxidative stress) increases slowly and increases marginally during initial days of postpartum period [4]. Metabolic demands during transition period increase the concentration of reactive oxidative species and immune cells are very sensitive to peroxidation [5]. Natural immunosuppression occurs in most cattle during transition period, exaggerated by the factors like negative energy balance, hypocalcemia, and increased cortisol level around calving [6]. The balance to maintain the levels of peroxidation are done primarily by antioxidants viz. catalase and superoxide dismutase (SOD). Therefore, estimation of their concentration and imbalances during this period provides affirmation of tissue or membrane damage.

Dairy cattle during lactation period depend upon body reserve to meet their demands but a prolong negative energy imbalance (NEB) is associated with the development of ketosis [7]. The subclinical form of ketosis is a common condition seen in high producing dairy cattle (SCK; 1.2 to 2.9 mmol of beta-hydroxybutyric acid (BHBA)/L of serum) [8]. During the period of negative energy balance key hormone expression and tissue responsiveness alter to increase lipolysis and decrease lipogenesis, causing an increase in blood levels of non-esterified fatty acids (NEFA) and BHBA [9]. Concentration of liver fat after calving is an indicator of fat mobilization and may affect hepatic gene expression of enzymes involved in gluconeogenesis and fatty acid oxidation [10]. Genes associated with β-oxidation of fatty acids are carnitinepalmitoyl-transferase 1A (*CPT1A*); acyl-CoA synthetase, long chain (*ACSL1*) and acyl-CoA dehydrogenase, very long chain (*ACADVL*). Data related to response of buffaloes to the changes in metabolic demand during this period have been documented by many authors [11,12] in the form of various hemato-biochemical and oxidative parameters. Only a low incidence level of metabolic diseases has been documented in buffaloes as compared to other ruminants. However, some authors [13,14] still consider ketosis/negative energy balance as one of major metabolic disease in buffaloes during lactation. The concentration of blood metabolites like glucose, BHBA and insulin provides some information of metabolic status of the buffalo and can be used as a surveillance tool [15]. Ketosis can be monitored using blood, urine, or milk samples from transition buffaloes [16]. Urine or milk testing in a herd periodically is the easiest and cost-effective way for qualitative detection of ketone bodies but plasma BHBA concentrations and changes in mRNA expressions of selected markers associated with negative energy balance provides better understanding of dynamic regulation of metabolism during this period [17].

Despite having documented data of various biochemical markers used as indicators of ketosis/negative energy balance condition in dairy cattle and buffaloes, genomic technologies may help in understanding of the involvement of different metabolic pathways of liver that are sensitive to nutrient partitioning and balancing during transition period. The hypothesis tested was that the concentrations of blood metabolites and mRNA expressions of selected markers indicative of negative energy balance are different between time points during transition period.

## MATERIALS AND METHODS

### Animals, diet and experimental design

A total of 210 she buffaloes during their transition period (−30 to +30 days) were selected randomly from dairy farms and the veterinary referral hospital, Faculty of Veterinary Sciences and Animal Husbandry, Sher-e-Kashmir University of Agricultural Sciences & Technology of Jammu, R.S. Pura, UT of Jammu & Kashmir, India. Animal experiment was approved by the Committee for the Purpose of Control and Supervision of Experiments on Animal (CPCSEA), New Delhi (No.: 25/15/2018/CPCSEA). These buffaloes were categorized into three groups, on the basis of days of their transition period: Gp-I (−30 days), Gp-II (near parturition), Gp-III (+30 days). Buffaloes selected for this study had a body weight of 724.00 kg (±60), previous lactation length of 286 days (±7) previous lactation milk yield 1,800 to 2,058 kg (±125), body condition score of group-I; 3.04 (±0.04), group II; 2.914 (±0.038); and group III; 2.8 (±0.029) and with parity between 3rd-6th. The buffaloes were given stall feeding (containing 25 to 30 kg of dry fodder, salt lick and *ad libitum* water provided.

### Sample collection

Blood samples were collected from each buffalo of all the groups on pre-decided days (a month pre-partum, close to partum and one-month post-partum) in ethylenediamine tetra-acetic acid (EDTA) contained vacutainer; VACUETTE (Cat. 455036, Greiner bio-one, Austria) for extraction of total ribonucleic acid (RNA) from peripheral blood mononuclear cells and in serum clot activator vacutainers; VACUETTE (Cat. XLGA-C5; Greiner Bio-One India Pvt. Ltd., Noida, India) for estimation of BHBA levels using enzyme linked immunosorbent assay (ELISA) kits (Immunotag; GBiosciences, St Louis, MO, USA). Blood samples collected in heparin containing vacutainers VACUETTE (Cat. 455051; Greiner bio-one, Austria) were used for estimation of oxidative biomarkers; glutathione peroxidase (GPX), LPO, SOD, and catalase. Buffaloes in mid-lactation period were considered a control group for estimation of differential gene expression of interest. Glucose concentration in whole blood (mg/dL) was estimated with Accu-check glucometer immediately with fresh blood.

### Urine collection and analysis

Urine samples from the buffaloes were collected in sterile Uricol (Cat. PW016; Himedia, Mumbai, India). Then urine analysis strips (SD Urocolor, Standard diagnostics, INC, Kyonggi, Korea) were dipped in urine for seconds and compared with corresponding color chart on the bottle label and results were read immediately. Immediately after collection, Rothera’s test was performed with 1 to 2 gm of ammonium sulphate added in 5 mL of urine, 5% solution of sodium nitroprusside and concentrated ammonium hydroxide was layered over the sample to obtain purple ring within 2 to 3 min.

### RNA extraction from whole blood and its gel documentation

Total RNA was extracted from EDTA preserved blood by trizol method (Invitrogen Life Technologies, Carlsbad, CA, USA). Trizol reagent (Cat. 15596018; Thermo Fisher Scientific, Ahmedbad, India) which contains various components viz guanidine isothiocyanate, phenol and isoamyl which facilitate the extraction of RNA with high yield. EDTA contained blood was diluted in phosphate buffer solution (PBS) and then layered over the HiSep LSM-1077 (Cat. LS001; HiMedia, India). Subsequent centrifugation at 2,400 rpm for 24 min at 4°C was done in a refrigerated centrifuge (Centrifuge 5430R; Eppendorf India Ltd., Chennai, India). After separation of buffy coat in 1× PBS and its centrifugation at 17,800 rpm for 10 min thrice, a clear pellet was obtained at the bottom. TRIzol (Cat. 15596026; Thermo Fisher Scientific, India) was added after separation of supernatant and mixed properly with pellet. After a 30 to 40 min. at −20°C incubation period a TRIzol, chloroform (Cat. MB109; Himedia, India) and isoproponalol treatment was applied to the pellet. The pellet was subsequently washed three times with ethanol and collected by centrifuging at 7,500 rpm for 5 min. Following air drying of the pellet, 20 μL of diethyl pyrocarbonate (DEPC) treated water (Cat. R0601; Thermo Fisher Scientific, India) was added. Optical density (OD) of the final pellet was taken spectrophotometrically (Biospectrometer; Eppendorf India Ltd., India) to determine concentration and purity. Cut-off point of RNA concentration (μg/mL) recommended for cDNA synthesis was >1.0 μg/mL with ratio of 260/280 nm >1.5. Gel electrophoresis with 1% agarose of molecular grade containing 1 μg/mL of Ethidium bromide (Cat. H5041; Promega, New Delhi, India) was made with 1× TAE (Tris-acetate-EDTA) buffer (40× molecular grade, Cat. V4281; Promega, USA) treated in DEPC water (Cat. MB076; Himedia, India). Voltage 1 to 5 V/cm was applied across the gel until the bromophenol blue migrated to appropriate distance. Resultant band (28s band and 18s) in the gel was seen with gel documentation system (Vilber; Eppendorf India Ltd., India), under the illumination of UV light and confirmed the presence of RNA.

### cDNA synthesis and its validation

Total isolated RNAs were subjected to cDNA synthesis using Hi-cDNA synthesis kit (Cat. MBT076-100R; Himedia, India). A reaction mixture of 1 μg of RNA template, 2 μL of oligo-dtprimer (oligonucleotides that contained a segment of repeating deoxythymidines) (10 pmol) and 7 μL of nuclease free water in sterile polymerase chain reaction (PCR) tubes was made. This mixture was incubated for 5 min at 65°C in a thermal cycler (BioRad T100 Thermal cycler, Gurugram, India). Other components: 4 μL of RT buffer, 2 μL of 10× solution, 10 mMdNTP (Deoxynucleoside triphosphates) solution, 0.5 μL of ribo-nuclease inhibitor and 1 μL of reverse transcriptase enzyme were added in template RNA and primer mixture to make a final volume of 20 μL. This mixture was subjected to PCR conditions; one cycle of 42°C for 60 min, 70°C for 5 min and hold at 4°C in a thermal cycler. A negative control reaction mixture was made, containing all the kit components except RNA template.

Glyceraldehyde-3-phosphate dehydrogenase (GAPDH) was used as a house keeping gene for validation of cDNA; forward primer (AAGGCCATCACCATCTTCCA) and reverse primer (CCACTACATACTCAGCACCAGCAT). The reaction mixture totaled 20 μL containing 10 μL of PCR pre-mix (Thermo Scientific, India), 2 μL of cDNA, 0.3 to 0.5 μL of GAPDH forward and reverse primer (10 pm each) and nuclease free water to make the final volume in sterile PCR tubes. These reactions were subjected to PCR cyclic conditions and gel electrophoresis for the validation of cDNA products when compared with negative control (Table 1).

### Microarrays and quantitative real-time reverse transcription polymerase chain reaction

#### In-silico analysis of molecular markers for ketosis

i) Datasets and their normalization for analysis of gene expression in ketosis: In order to identify the molecular markers, gene expression datasets of 14 samples of normal liver of healthy cows of early postpartum and 14 samples from cows in early postpartum ketosis were retrieved (GEO ID: GSE4304; UIUC *Bostaurus* 13.2K 70-mer oligoarray) and analyzed by a variety of bioinformatics tools. All samples from healthy non-diseased cows were considered as controls (Non-ketosis), their samples were considered as experimental or test group. The expression file was subjected to RMA algorithm to make the samples comparable. Expression values were computed based on corresponding probe set annotations. Alt Analyze python scripts were used to call the Affymetrix Power Tools, distributed with the GPU license [18].

### Gene expression and clustering analysis

The analyses of gene expression levels and gene ontology (GO) annotation were carried out with GO-Elite software, using default options [19]. Microarray expression values were reported as log2 values. Differentially expressed genes were identified using a combination of a >2-fold change in expression with a statistical significance of p<0.05 (moderated t-test) using Benjamini-Hochberg correction method [20]. Differentially expressed genes were subjected to hierarchical clustering to identify gene clusters within the control and test groups [21]. Gene-set enrichment analysis and comparison was executed using the GO-Elite in AltAnalyze, where only terms with an false discovery rate adjusted enrichment p< 0.05 was considered for further evaluation.

### Identification of ketosis specific markers

To identify tissue/cell markers in both ketosis and healthy samples, we first filtered the genes with expression level of >90 percentile of all samples, and then we compared the abundantly expressed genes in both ketosis and non-ketosis samples to identify both common and uniquely expressed genes. A gene was defined as enriched in tissue ‘X’ if the average expression of the gene in tissue ‘X’ was at least 3 times greater than its average expression in all other 66 tissues. We defined a tissue-specific gene marker as the gene not only enriched in tissue ‘X’, but also expressed highest in tissue ‘X’ and when expression in tissue ‘X’ was at least 1.5 times higher than its expression in any other tissues. Further, Marker Finder algorithm in Alt Analyze was performed within each independent dataset to derive putative cell-population-specific markers for gene-set enrichment [22].

### Quantitative real time PCR for quantification of expression of gene interest

Real time PCR helps in the amplification of the targeted molecule in real time. It was conducted by quantitative real time PCR cycler- MyGo mini (MyGo 002). A reaction mixture with 10 μL Hi-SYBr master mix (Cat. MBT074-100R; Himedia, India), 0.5 μL of forward and reverse primer of gene of interest and nuclease free water up to 20 μL volume was made and subjected to qRT-PCR reaction condition (Table 2). For calculation of Cq values (quantification cycles), comparative CT method also referred to as the 2^−ΔΔCt^ method was used with internal gene control; GAPDH and buffaloes in mid-lactation as control group [23].

### Oxidative stress markers

Various oxidative stress markers such as SOD, GPx, LPO, and catalase were evaluated from transition buffaloes (Group-I, II, and III). For evaluation, 5 mL of blood in heparin containing vacutainer was taken and centrifuged at 3,000 rpm for 10 min. After that, plasma was stored and left over red blood cells (RBC) lysate was used to determine the antioxidant status. Washing of RBC lysate was done with normal saline by diluting in a ratio 0f 1:1 and centrifuged for 10 min at 3,000 rpm. Washing was done thrice. 1% lysate (100 μL of lysate and 990 μL of distilled water) and 33% lysate (330 μL of lysate and 640 μL of PBS with pH 7.4) was prepared.

### Lipid peroxidation

Lipid peroxidation in erythrocytes was determined by the evaluation of malondialdehyde (MDA) production by the method of [24]. In brief, 1 mL of 33% RBC haemolysate was taken and added to 1 mL of 10% w/v of trichloracetic acid. After thorough mixing, mixture was centrifuged at 3,000 rpm for 10 min and supernatant was extracted. To 1 mL of the extracted supernatant added 1 mL of 0.67% w/v of thiobarbituric acid and kept in water bath for 10 min, cooled and diluted with 1 mL of distilled water. The same reagents were used except the haemolysate for control samples. Absorbance was noted at 535 nm in the spectrophotometer (Biospectrometer; Eppendorf India Ltd., Country).

### Calculation

Calculations were done using the extinction co-efficient (EC, 13,100 M^−1^cm^−1^) and results expressed in mM MDA per mL of blood, using the following formula.


$$\text{LPO }\left(\text{mM}\frac{\text{MDA}}{\text{mL}}\right)=\frac{\text{OD}}{\text{EC}}\times \frac{\text{Total volume of reaction mixture}\times 1,000\times \text{DF}}{\text{Amount of sample taken}}$$

### Catalase

The plasma catalase activity was determined by the method described by Marklund and Marklund [25]. In brief, 2 mL of phosphate buffer along with 20 μL of 1% lysate were taken. This mixture then incubated with 1 mL of 30 mM of hydrogen peroxide at 37°C and the decrease in the absorbance was observed at every 10 s interval for 1 min at 240 nm OD. The catalase activity was expressed as μmoles of H_2_O_2_ utilized/min/mg Hb using 36 as molar extinction coefficient of H_2_O_2_.

### Superoxide dismutase

The activity of erythrocytic SOD was determined by method of Hafeman et al [26]. In brief, 1.5 mL of 100 mM tris HCl buffer, 20 μL of 1% haemolysate, 0.5 mL of 6 mM EDTA and 1 mL of pyrogallol was added. The rate of auto-oxidation of pyrogallol was taken from the increase in absorbance at 420 nm in a spectrophotometer, every minute after a lag of 30 s up to 4 min. For the test, an appropriate amount of enzyme was added to inhibit the auto-oxidation of pyrogallol to about 50%. A unit of enzyme activity is defined as the amount of enzyme causing 50% inhibition of the auto-oxidation of pyrogallol observed in blank. The activity of SOD was expressed as SOD units/mg protein.

#### Calculation


$$\text{SOD activity }(\text{U}/\text{mg of Hb})=\frac{\left(\Delta \text{E}0-\Delta \text{E}\right)}{\Delta \text{E}0}\times \frac{1}{2}\times \frac{1}{\text{g of protein in }0.01\;\text{mL}}$$

ΔE_0_ = change of absorbance of pyrogallol; ΔE = change of absorbance of sample.

### Glutathione peroxidase

The activity of GPx in erythrocytes was performed as per method of Loor [27]. In brief, 0.1 mL of erythrocytes was taken. To this was added 1 mL of glutathion peroxidase, 1 mL of 0.4 M sodium phosphate containing EDTA, 0.5 mL of 0.01 M of NaN_3_ and distilled water to make a volume of 5 mL. The solution was incubated for 5 min. After the incubation, 1 mL of 125 mM hydrogen peroxidase added and r incubation continued for about 3 min. 4 mL of meta-phosphoric acid was added to 1 mL of liquid from the incubation mixture. Then, 2 mL of this solution was added with 2 mL of 0.4 M sodium hypo-phosphate (NaHPO_4_) and 1 mL of DTNB (5,5’-dithio-bis-2-nitrobenzoic acid) reagent. For the blank solution, water was used instead of hydrogen peroxidase and rest of the procedure was same. OD was taken at 412 nm against blank solution.

#### Calculation


$$\text{Activity of GPx}=10\;\text{log}\frac{\text{Co}}{\text{C}}$$

### Statistical analysis

All statistical analyses were performed using SPSS (Statistical Package for the Social Sciences) statistical software version 16 (IBM SPSS Statistics 16). Data were analyzed using analysis of variance and the Duncan’s multiple range test was performed to provide significance levels (p< or >0.05) for the difference between the means.

## RESULTS

### Urine analysis for detection of ketone bodies

**Figure 1:**
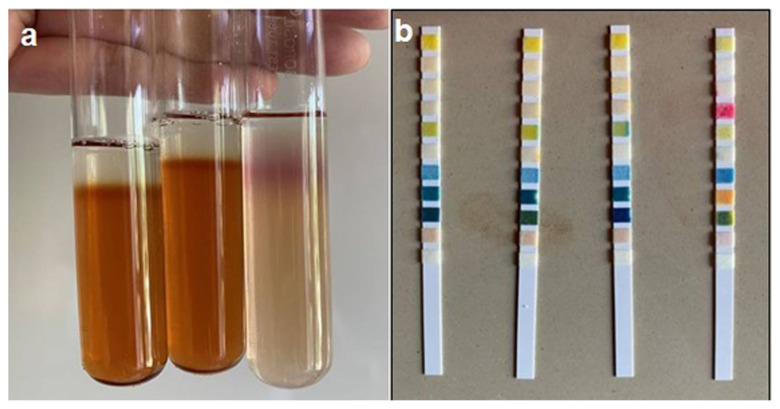
Urine analysis (a) Rothera’s test: Negative Rothera’s test result of urine samples with positive control in third test tube; (b) SD Urocolor strips for detection of ketone bodies: negative result of ketone bodies detection in urine strips (SD Urocolor) in first three strips from left to right with positive control (4th block with pink colour).

Urine sample analysis of the selected buffaloes showed negative result for Rothera’s test and urine strips (SD Urocolor) for ketone bodies when compared with their positive control (Figure 1).

### Metabolic measurements

**Figure 2:**
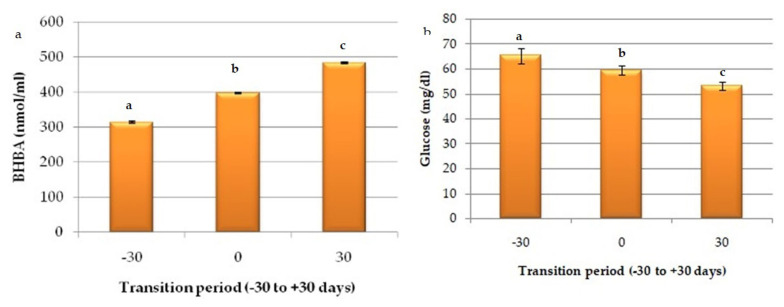
Graphs depicting trends of (a) beta-hydroxy butyric acid (BHBA, nmol/mL) and (b) glucose (mg/dL) during transition period (GP-I, -30 days; GP-II, near parturition; and GP-III, +30 days) in buffaloes. ^a–c^ Means with different superscripts between rows differs significantly (p<0.05).

Serum level of BHBA (nmol/mL) increased significantly (p<0.05) from pre-partum to post-partum period; Gp-I to Gp-III (313.96±2.81 nmol/mL, 397.36±1.69 nmol/mL, and 483.69±1.98 nmol/mL, respectively) and glucose concentrations showed significant decline (p<0.05) from far off dry period to early lactation (Gp-I, 65.31±3.01; Gp-II, 59.44± 1.78; and Gp-III, 53.08±1.74), although buffaloes in these groups were non-ketotic (Figure 2).

### Differential expression of genes by microarray assay and qRT-PCR

#### In-silico analysis for ketosis markers

**Figure 3:**
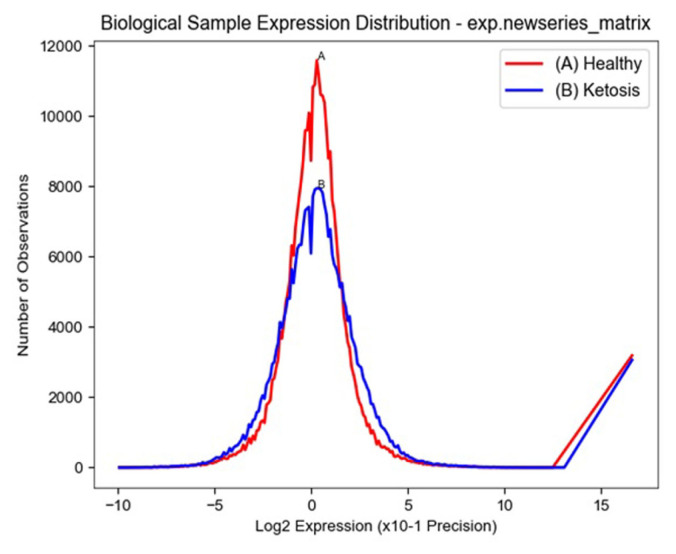
Distribution of microarray probe intensities in ketosis and healthy samples.

The distribution of the probe intensities varied from 0 to 8,000 for ketosis and 0 to 12,000 observations for healthy samples (Figure 3).

### Differentially expression analysis, clustering, and principal component analysis

**Figure 4:**
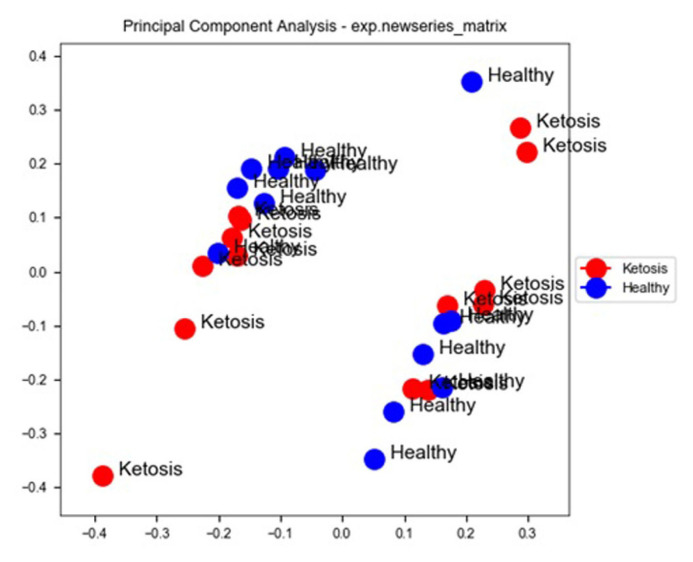
Principal component analysis (PCA) of ketosis and healthy samples sharing different principal components. Red spheres represent ketosis samples whereas blue spheres represent healthy samples.

**Figure 5:**
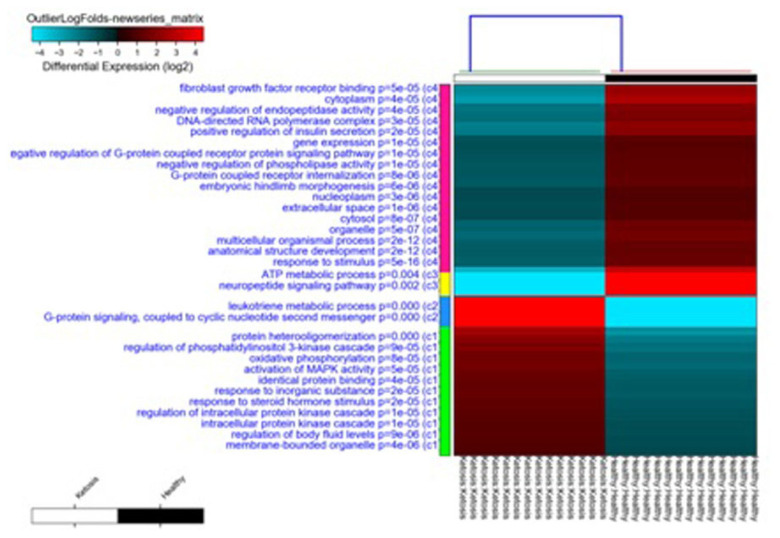
Heatmap showing the log-fold changes of genes and hierarchal clustering of various Ketosis and Healthy samples.

Compared with normal non-ketosis samples, 2,121 genes were found to be differentially expressed in the ketosis samples. Among these 1,053 were found to be upregulated and 1,068 were found to be downregulated. Principal component analysis of the two groups revealed that all ketosis samples were sharing one component, and all healthy samples were sharing other principal components along with the heat map showing log fold changes of gene and hierarchal clustering of various healthy and ketosis samples (Figures 4 and 5).

### Gene ontology and pathway analysis

**Figure 6:**
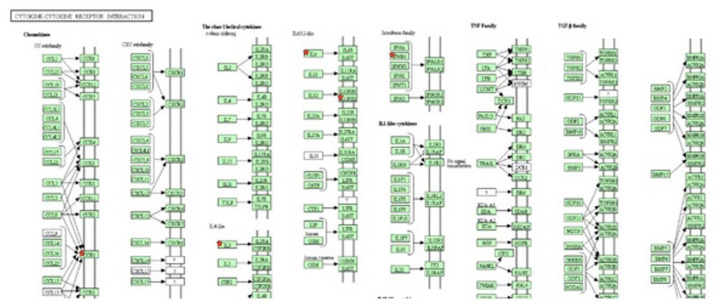
Participation of up-regulated genes in cytokine receptor signaling pathways. Genes in star marked were found to be up-regulated in ketosis samples.

**Figure 7:**
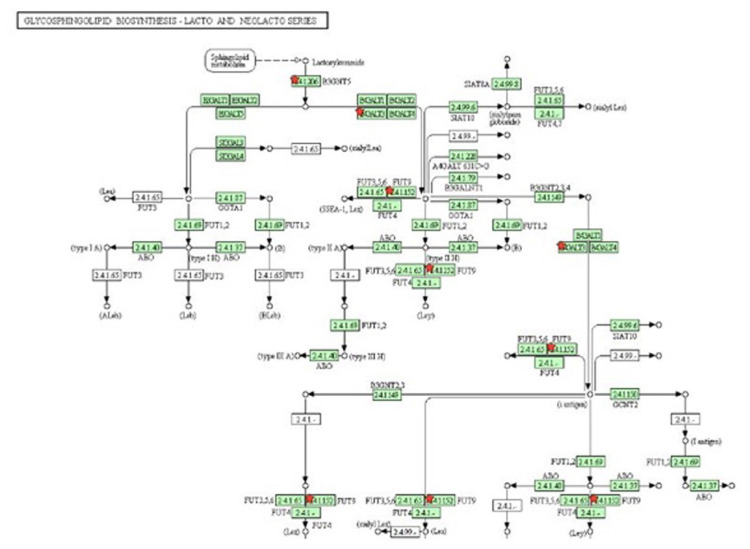
Participation of down-regulated genes in glycosphingolipid biosynthesis. Genes in star marked were found to be down-regulated in ketosis samples.

Gene ontology analysis revealed that some of the genes were directly related to the immune response, inflammatory response, and chemotaxis and leukocyte cell-cell adhesion. Upregulated genes included interleukin-6 (*IL6*), interleukin-3 (*IL3*), C-C chemokine receptor type 1 (*CCR1*), interferon beta 1 (*IFNB1*), acetylserotonin O-methyltransferase like (*ASTML*), interleukin-12 receptor, beta 1 (*IL12RB1*), and leptin (*LEP*). Pathway analysis of the up-regulated genes was related to the immune response, with most genes involved in the cytokine-cytokine receptor interaction (Figure 6), induction, regulation of the local inflammatory response and oxidative stress related mechanisms. The group of down-regulated genes in the ketosis guanylate cyclase activator 1B (*GUCA1B*), general receptor for phosphoinositides-1 (*GRTP1*) found to involve in Glycosphingolipid biosynthesis (Figure 7), fat digestion and absorption pathways and in fatty acid βoxidation.

### Marker genes identification for ketosis

**Figure 8:**
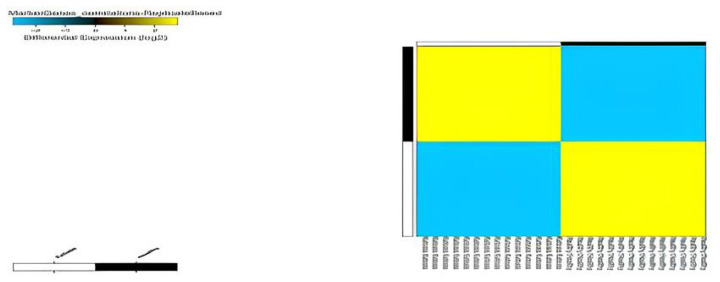
ICGS analysis of transcriptomes for finding markers of ketosis and health.

A total of 60 ketosis- specific genes were determined by Pearson correlation coefficient. The top 10 correlated genes determined with at least a 0.3 Pearson correlation coefficient were selected as the gene signature for each cell cluster (Figure 8). The top 20 genes identified as markers in Ketosis include *IL6*, *IL3*, *CCR1*, *IFNB1*, *IL12RB1*, *ASTML*, and *LEP*. On the other end, the top 10 genes identified as marker genes in healthy samples were beta-1,3-N-acetylglucosaminyltransferase (*B3GNT5*), beta-1,4-galactosyltransferase (*B4GALT3*), and fucosyltransferase 9 (*FUT9*) (Table 3).

### qRT-PCR analysis

Out of 20 potential markers of ketosis, two were selected (ASTML; upregulated and GUCA1B; downregulated) and analysed by qRT-PCR technique (Table 4). Additional two sets of genes (*CPT1A*; upregulated and *IGF-1*; downregulated) that were not a part of microarray assay were also subjected to qRT-PCR cycling condition (Table 4). They have role in β-oxidation during post-partum stimulation of hepatic fatty acid oxidation to maintain energy demand via gluconeogenesis [28] and uncoupling of growth hormone-insulin growth factor-1 (GH-IGF-1) axis causing downregulation of liver growth hormone receptor-1A (GHR 1A) and IGF-1 level, ultimately elevation in the growth hormone concentration that antagonizes the function of insulin and causes lipolysis and gluconeogenesis in early lactation [29] respectively.

**Figure 9:**
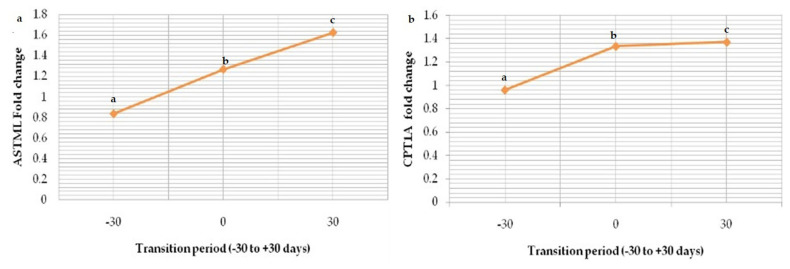
Graphs depicting expressions of upregulated genes during transition period (GP-I; −30 days, GP-II; near parturition and GP-III; +30 days) in buffaloes; (a) *ASTML* (b) *CPT1A*. ^a–c^ Means with different superscripts between rows differs significantly (p<0.05).

**Figure 10:**
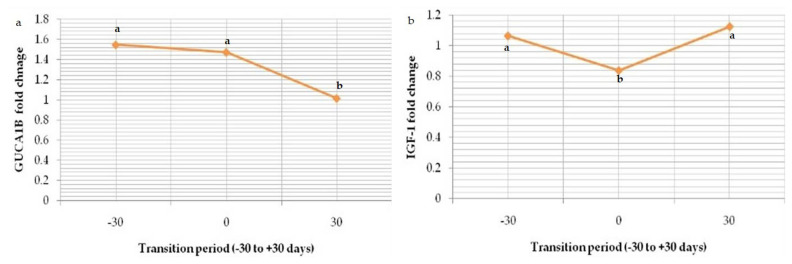
Graphs depicting expressions of downregulated genes during transition period (GP-I, -30 days; GP-II, near parturition; and GP-III, +30 days) in buffaloes; (a) *GUCA1B* (b) *IGF-1*. ^a–c^ Means with different superscripts between rows differs significantly (p<0.05).

Relative mRNA expression of marker ASTML showed significant (p<0.05) upregulation as transition period progressed from prepartum period (Gp-I) to calving (Gp-II) and post-partum period (Gp-III); 0.836±0.02, 1.268±0.03 and 1.624±0.01 respectively (Figure 8). The same trend in the expression level of CPT1A was seen from Gp-I to Gp-III (0.96±0.06, 1.336±0.02, and 1.372±0.02) (Figure 9). Whereas downregulation in the expression level of GUCA1B was seen from pre-partum period (1.548±0.05) to calving (1.474± 0.08) and post-partum period (1.014±0.04) (Figure 10). The expression fold of IGF-1 marker showed a significant (p<0.05) trend of downregulation from pre-calving (1.063±0.05) to near calving (0.839±0.02) term thereafter, gradual increase in the concentration as post-calving period progressed (1.123 ±0.02).

### Changes in oxidative stress markers during transition period in buffaloes

The imbalance between oxidative marker and antioxidant markers level causes oxidative stress. On analysis of these antioxidant and oxidative markers, our results showed a significant difference (p<0.05) amongst these groups providing sufficient evidence about oxidative biomarkers and antioxidants production during transition period in buffaloes.

Our study showed significant (p<0.05) increase in the mean±standard error (SE) values of MDA (nmoles of MDA produced/g of Hb/h) in Group-II (near parturition) with comparison to Group-I (−30 days) and Group-III (+30 days). Whereas, mean±SE values of SOD (U/mg of Hb) and catalase (CAT; μmoles) showed a significant decrease (p<0.05) in their values from Group-I (−30 days) to Group-III (+30 days) (Table 5). Blood GPx (mg/Hb) levels showed trend of significant decrease (p<0.05) from pre-partum period (−30 days) to near parturition time. Although, a significant increase (p<0.05) in the levels of GPx was seen in buffaloes when transition period progressed to +30 days (Table 5).

## DISCUSSION

Energy and nitrogen demand by the conceptus in late pregnancy are mostly met by dam’s glucose and amino acid uptake by the placental tissues. During postpartum period there is marked increase in mammary demands of glucose, amino acids and fatty acid. Therefore, rates of hepatic gluconeogenesis and adipose tissues are greatly increased. Bovine liver has limited capacity to metabolise NEFA into triacylglycerol (TAG) which accumulates in the liver as threshold crosses the limit. Thereafter, acetyl CoA resulting from oxidation of fatty acid are not utilised by the tri-rcaboxylic acid (TCA) cycle and later converts into acetone, acetoacetate and BHBA which appears in milk, urine and blood. The free fatty acid and BHBA are considered as indicators of negative energy balance and peak concentrations are seen in the early-postpartum period as reported by Bryers [30]. In the present study, the values of BHBA increased from pre-partum to post-partum period but under the normal range of sub-clinical ketosis (<1,200 nmol/mL) in all the three groups. Low prevalence of metabolic diseases such as ketosis can be partially explained with ability of buffering and breakdown of carbohydrates (non-structural), low thyroid activity and retention of minerals [31]. In the present research study, transient decrease in blood glucose concentration from the far-off dry period up to the early lactation was observed. Our results are in accordance with Ambrosio et al [32] suggesting severe negative energy balance characterised by reduced blood glucose and insulin levels.

In-silico analysis of selected potential markers of ketosis/negative energy balance viz; ASTML (upregulated) and GUCA1B (downregulated) validated with the qRT-PCR analysis provides a similar trend in fold expression of ASTML showing upregulation throughout the transition period and GUCA1B giving a downregulated trend. As pathways involved in the upregulation and downregulation of selected markers are cytokine-cytokine interaction, oxidative stress pathways and Glycosphingolipid biosynthesis, respectively. These results suggest that negative energy balance reduces the expression of genes involved in fat biosynthesis and metabolism with upregulation in pro-inflammatory cytokine interaction and free radical stress at molecular level. However, further research is needed to verify whether these genes influence the total amount of fat secretion in the milk or instead influence the fat percentage in the milk.

Similarly, the fold change in expression level of CPT1A in our study showed significant upregulation (p<0.05) as buffaloes progresses form dry period to early lactation (3 weeks pre-partum to 3 weeks postpartum). Our results were in congruence with the study of Singh et al [33]. Involvement of CPT1A in stimulation of hepatic fatty acid β-oxidation to maintain energy demands in the hepatic tissues especially for production of glucose from reserve sources can partially explain its upregulation during this phase [34]. Expression fold of IGF-1 in our study has showed a trend of downregulation from pre-calving to immediate calving period and thereafter, a gradual increase was observed during early post-partum period. The down regulation of IGF-1 indicates a relationship of insulin with GHR 1A in liver as GHR 1A is controlled by insulin and growth hormone (GH) in turn controls IGF-I synthesis and secretion. Therefore, reduced negative feedback on GH by IGF-1 promotes lipolysis, which releases NEFA into blood. The gradual increase in the expression fold of IGF-1 during post-partum period could be an attribute of recovered growth hormone resistance by the hepatic tissue that occurs during this period [35]. However, in some studies suppressed IGF-1 synthesis has been observed in animals having evident negative energy balance [36]. Our study model focuses on transition period as majority of metabolic diseases occur around this time and provides scope for nutritional interventions that can have profound effect on prevention of clinical and subclinical forms of production diseases.

Oxidative stress occurs during the period of high metabolic demands which leads to an imbalance of anti-oxidant defense markers and reactive oxygen species. The reactive oxygen species progressively increased from late lactation to post parturient period, even higher in peak lactation [37]. In our study we found significant increase (p<0.05) in level of LPO during calving and postpartum period and decreased level of SOD, GPx, and catalase. The adaptation to negative energy balance during transition period in various tissues, primarily NEFA leads to oxidation in an intensified manner which eventually results in the formation of reactive oxygen species [38]. The intermediate product of lipid peroxidation, MDA level is increased during this period suggesting a higher level of lipid peroxidation. Superoxide dismutase is considered as the first line of defense against oxidative stress radicals, by converting superoxide radical to hydrogen peroxide and is considered as important antioxidant marker. A significant decrease in SOD is evident of higher oxidative stress during periparturient period in dairy cattle [39]. Glutathione peroxidase plays an important role in antioxidant defense mechanism by reducing the hydrogen peroxide into water and alcohol. Glutathione is used as a reducing agent in the reaction [40]. From the level of GPx during calving and immediate post- partum time, one can say that animals are having a marked reduction in antioxidant markers and an increase in reactive oxygen species during transition period. Also, In-silico analysis revealed pathways regulated by upregulated genes are cytokine interactions causing inflammatory response along with oxidative stress related to significant increase in free radical concentrations at molecular level. This enables us to consider these putative biomarkers for prompt detection of occurrence of metabolic imbalances during transition period in buffaloes. It ultimately provides scope of nutritional or therapeutic interventions to prevent their occurrence.

Physiological conditions associated with insufficient energy supply predispose dairy animals to metabolic and microbial diseases. The role of inflammatory mediators in the decline in fertility/production is not known, given the range of various physiological processes involved. An improved understanding at the molecular level plays an important role in normal immune function, metabolism, and reproduction. This may improve our ability to predict and prevent transition disorders. Therefore, a scientific approach for the early diagnosis of the metabolic and production disease in dairy animals is required. We have integrated the evidence from in-silico analysis, qRT-PCR validations of potential markers along with putative oxidative stress indicators of ketosis/negative energy balance in buffaloes that can be used for earliest diagnoses as they explain the complex physiological processes in the liver and adipose tissue that are elicited by the plane of nutrition during the periparturient period.

## CONCLUSION

In conclusion, bioinformatics analysis of high-throughput gene expression revealed the involvement of multiple pathways, including the inflammatory pathway, fatty acid pathway and cytokine interaction pathway during negative energy balance in transition period. The experimental analysis reveals that molecular biomarkers and oxidative stress indicators may act as potential biomarkers for the early detection of negative energy balance. Furthermore, we should consider other clinical diseases or signs to correlate to improve the accuracy of diagnoses.

## Figures and Tables

**Table 1:** PCR cycling condition for validation of cDNA with GAPDH primer

Step	Temperature (°C)	Time	Cycle
Initial denaturation	94	3 min	1
Denaturation	94	30 s	35
Annealing	58.9	30 s	35
Extension	72	45 s	35

PCR, polymerase chain reaction; GAPDH, glyceraldehyde-3-phosphate dehydrogenase.

**Table 2:** qRT-PCR cycling conditions

Steps	Temperature (°C)	Cycles	Time
Pre incubation	95	1	5 min
Amplification (3 steps)	95	40	20 s
Annealing	Varied by gene of interest	40	15 s
	72	40	15 s
Pre melt	95	1	5 s
Melting	70	1	1 min
Melting	95	1	40 s
Cooling	40	1	30 s

qRT-PCR, quantitative real time-polymerase chain reaction.

**Table 3:** Potential marker genes for detection of bovine ketosis

Gene symbol/ ID	Pearson rho	Pearson p-value	Cell state
*LOC510604*	−4.55E-05	0.9998167	Healthy
*GUCA1B*	−1.30E-04	0.9994769	Healthy
*MGC151578*	−1.59E-04	0.9993597	Healthy
*4031*	−1.65E-04	0.9993331	Healthy
*LOC784882*	−1.88E-04	0.9992407	Healthy
*18181*	−2.06E-04	0.9991703	Healthy
*7889*	−2.19E-04	0.9991167	Healthy
*GRTP1*	−3.19E-04	0.9987156	Healthy
*18929*	−3.23E-04	0.9986973	Healthy
*728*	−3.80E-04	0.9984709	Healthy
*14241*	−8.45E-06	0.9999659	Ketosis
*ASMTL*	−5.31E-05	0.9997861	Ketosis
*21265*	−6.18E-05	0.9997509	Ketosis
*20145*	−6.53E-05	0.9997369	Ketosis
*SNCA*	−7.23E-05	0.9997085	Ketosis
*AEBP1*	−8.36E-05	0.9996631	Ketosis
*MGC155209*	−1.24E-04	0.9994985	Ketosis
*LOC508367*	−1.40E-04	0.9994373	Ketosis
*CLPP*	−1.53E-04	0.9993812	Ketosis
*MGC160122*	−1.59E-04	0.9993588	Ketosis

*GUCA1B*, guanylate cyclase activator 1B; *GRTP1*, growth hormone regulated TBC protein 1*; ASMTL, a*cetylserotonin O-methyltransferase like; *SNCA*, acetylserotonin O-methyltransferase like*; AEBP1*, AE binding protein 1; *CLPP*, caseinolytic mitochondrial matrix peptidase proteolytic subunit.

**Table 4:** Primer sequences and annealing temperatures of genes of interest

Genes	Annealing temperature (°C)	Size (bp)	Sequence
*CPT1A*	58.7	292	F:TTATGTGAGCGACTGGTGGG
			R:GTGCTGGATGGTGTCTGTCT
*ASTML*	67.3	215	F:CTCATTCGCCACCCCGCA
			R:CGCTGTGCTCCTTCCCAC
*GUCA1B*	65.5	253	F:CTCTGCTCCCTGCCGTCC
			R:GGTGTTGTCCCCATTCGTGT
*IGF-1*	59.6	140	F:CATCCTCCTCGCATCTCTTC
			R:GAAATAAAAGCCCCTGTCTCC

*CPT1A*, carnitinepalmitoyl-transferase 1A; *ASTML*, acetylserotonin O-methyltransferase like; *GUCA1B*, guanylate cyclase activator 1B; *IGF-1*, insulin growth factor-1.

**Table 5:** Oxidative biomarker and antioxidants level during transition period in buffaloes

Parameters	−30 days (Gp-I, n = 70)	0 day (Gp-II, n = 70)	+30 days (Gp-III, n = 70)
MDA (nmoles of MDA produced/g of Hb/h)	6.09^a^±0.10	7.01^b^±0.08	6.31^a^±0.13
CAT (μmoles of H_2_O_2_ utilised min^−1^ mg Hb^−1^)	125.89^c^±0.16	123.05^b^±0.14	119.30^a^±0.27
SOD (units/mg of Hb)	43.0^c^±0.39	39.10^b^±0.45	37.97^a^±0.36
GPx (mg/Hb)	3.16^c^±0.03	2.07^a^±0.02	2.21^b^±0.03

MDA, malondialdehyde; CAT, catalase; SOD, superoxide dismutase; GPx, glutathione peroxidase.

a–c Means with different superscripts between rows differs significantly (p<0.05).
